# Secondary Impacts of COVID-19 Pandemic in Fatigue, Self-Compassion, Physical and Mental Health of People with Multiple Sclerosis and Caregivers: The Teruel Study

**DOI:** 10.3390/brainsci11091233

**Published:** 2021-09-18

**Authors:** Lydia Giménez-Llort, Juan José Martín-González, Sara Maurel

**Affiliations:** 1 Department of Psychiatry and Forensic Medicine, Medical Psychology Unit, School of Medicine, Universitat Autònoma de Barcelona, 08193 Barcelona, Spain; 2 Institut de Neurociències, Universitat Autònoma de Barcelona, 08193 Barcelona, Spain; 3 ATUEM (Asociación Turolense de Esclerosis Múltiple), 44003 Teruel, Spain; info@atuem.es; 4 Department of Medicine, Hospital del Vall d’Hebron, Universitat Autònoma de Barcelona, 08035 Barcelona, Spain; saranieves.maurel@ehu.eus; 5 Department of Neuroscience, Psychiatry, Universidad País Vasco, 48940 Leioa, Spain

**Keywords:** secondary impact, COVID-19, multiple sclerosis, caregivers, fears, health, HRQoL, self-compassion, compassion, fatigue

## Abstract

The secondary impacts of the COVID-19 pandemic are distress triggers and risk factors for mental health. Conversely, self-compassion skills and compassionate thoughts/behaviors towards suffering may contribute to their alleviation. Both psychological constructs are interrelated in life-threatening diseases such as multiple sclerosis (MS). The Teruel Study retrospectively evaluated the impact of strict confinement on the 44 people with MS of this Spanish province and 24 caregivers, specifically assessing (1) fears and perceptions; (2) self-compassion (people with MS) and compassion (caregivers); (3) physical and mental health, and fatigue. Despite better housing conditions, people with MS considered confinement very difficult to handle, more than their caregivers, but they were less afraid of COVID-19 and worsening of MS. Still, they recognized worse health than before confinement. Reclusion and lack of walks were the worst of confinement. Caregivers also referred to lack of leisure and uncertainty–fear. All agreed the best was staying with the family, but some found ‘nothing’ positive. Self-compassion remained moderate–high and strongly correlated with their moderate levels of social function, vitality, physical role, and global health. Physical and cognitive fatigue scores were high, and self-compassion negatively correlated with them, explaining a 19% variance in global health. The high compassion of the caregivers did not correlate with any variable.

## 1. Introduction

Secondary impacts are defined as those caused by the COVID-19 pandemic indirectly, either through the effect of fear on the population or as a consequence of the measures taken to contain and control it [1]. The emergency and uncertainty associated with the COVID-19 pandemic accompanied by unsustainable economic losses and stigma have been identified as stressors and strong constraints for physical or emotional adaptation of individuals and society [2,3]. The emotional impact of COVID-19 has been described on groups ranging from medical staff to the broader population [4]. However, the fear of getting infected, fear of death, or worsening of health in those already ill and their caregivers can be strong triggers of emotional distress and risk factors for mental health problems [5]. Perceived stress levels may vary in each country, partly depending on the policy adopted in each territory, and can have important implications for the health and well-being of the population, especially in those already ill [6]. Poorer mental well-being (depression, loneliness, insomnia, daily life fatigue) during COVID-19 related to home confinement has been reported [7,8,9]. In the case of the Spanish population, six days after the WHO declared COVID-19 a global pandemic, a period of three months of strict confinement of the entire Spanish population was implemented. Afterward and still now, other governments adopt similar measures to counteract the fast spread of the pandemic despite severe confinement measures, quarantine, and social isolation exerting significant psychological, societal, and economic secondary impacts [7,8,9,10,11,12].

Conversely, compassion, the emotion that arises from recognizing the other’s suffering and in response to discomfort, contributes to alleviate suffering and create thoughts of empathy and feelings of affinity for those who suffer. Therefore, compassion is defined as the ability to establish sincere and empathetic connections with the suffering of others and to feel the desire to relieve their pain [13]. However, to connect with other people’s suffering and feel compassion for the “other,” it is necessary to start with oneself; that is, with self-compassion [14]. Thus, psychology conceives self-compassion as involving the mind and allowing the development of personal skills that also translate into interpersonal relationships to promote compassionate thoughts and behaviors [15]. According to Neff [15], self-compassion is defined as the ability to understand and support oneself in challenging moments, bearing one’s suffering with kindness and warmth as if it were an inward compassionate action and identifying what is needed to face this situation [16]. Compassion and self-compassion constructs are under strong feedback in those confronting a life-threatening disease or conditions such as multiple sclerosis (MS), and their interplay depends on many factors [16].

Multiple sclerosis is an autoimmune, neuroinflammatory, demyelinating, and neurodegenerative disease of unknown etiology, chronic in nature, and unpredictable in course that affects the central nervous system and the immune system [17,18]. Present throughout the world, MS can affect each person in a heterogeneous way, but it is ranked as the second leading cause of neurological disability in young adults, causing great functional and cognitive disability and detriment to their quality of life [17,18]. MS usually appears between the ages of 20 and 45 years and occasionally begins in childhood or later in life, with three-quarters of the patients being women. There is a large latitudinal difference in the distribution of MS, with higher figures in areas distant from the equator, so sun exposure and vitamin D deficiency are some of the most studied environmental risk factors [19]. Symptoms of MS vary depending on the size and location of central nervous system damage. They include mental (cognitive impairment, mood, emotional, and affective disorders) and physical health problems (decreased limb function, impaired bowel or bladder control, spasticity, sexual dysfunctions, vision disturbances, impaired balance, pain, and debilitating fatigue). Most importantly, the physical and mental effects can be synergistic. Thus, MS-associated fatigue can affect 87% of patients and cause significant physical, psychological, emotional, work, and social limitations. Forty percent of these patients consider fatigue as the most disabling symptom, and it constitutes one of the main causes of unemployment among people with MS. In fact, fatigue is defined as a subjective feeling of tiredness or lack of energy, disproportionate to the effort made or the degree of disability [20]. The pathophysiology of fatigue in MS is currently unknown. However, different hypotheses are proposed, and a multifactorial origin is postulated, combining different factors and a different specific weight in each of them [20]. It is important to emphasize that there are difficulties in understanding its pathophysiology, quantifying it, and treating it. In addition, many modifying MS treatments are based on suppressing or modifying the immune system, and therefore concerns about some MS medications increasing the chance of developing complications from a COVID-19 infection, as well as about clinical characteristics and outcomes in patients with COVID-19 and MS, have been raised during the pandemic [21,22,23,24,25,26].

The Teruel Study aimed to evaluate retrospectively, during June and July 2020, the physical and psychological impact of the COVID-19 pandemic on the 44 people with MS of this central-eastern area of the Iberian Peninsula and 24 caregivers. First, we questioned them about the features of their confinement, fears of worsening of the disease, being infected and ill with COVID-19, and the best/worst of that situation. After that, the study aimed to assess self-compassion (in people with MS) and compassion (caregivers) and relate it to physical and emotional health variables, specifically to fatigue. Questionnaires validated for the Spanish population that measure compassionate abilities and physical and mental health were used to determine the relationships and the predictive and explanatory validity of the psychological and emotional factors that make up self-compassion, considering states of general health, physical function, physical role, emotional role, social function, body pain, vitality, mental health, as well as physical, cognitive, and psychosocial fatigue.

## 2. Materials and Methods

### 2.1. Sample and Experimental Design

The sample population constituted 44 people with MS and 24 caregivers. The experiment had a cross-sectional, naturalistic design using consecutive cases diagnosed with MS according to the McDonald criteria [27,28]. These require objective evidence of at least two areas of myelin loss, or demyelinating lesions appearing in two distinct and time-spaced neurological areas, and a differential diagnosis excluding similar neurological diseases.

For the MS sample population, the inclusion criteria were: adults of 18 years or more from Teruel with a diagnosis of MS from the Specialized Health Service of Neurology by the Neurological Units and belonging to the Turolense Association of Multiple Sclerosis (ATUEM) or the Spanish Multiple Sclerosis Association (EME); sustained attention and verbal understanding of the language; participation and informed consent. The exclusion criteria were: refusal to participate in the study, or severe cognitive and/or physical impairment or disorder.

For the caregiver sample population, the inclusion criteria were: adults of 18 years or more who were caregivers of a person with MS participating in the Teruel Study, participation, and informed consent. The exclusion criteria were: refusal to participate in the study.

### 2.2. Procedures and Variables of Study

The research protocol, informed consent, and information collection instruments were reviewed and approved by the ethics committee of ATUEM. All participants were informed of the objectives of the present study and signed the informed consent before participating in the study. They also confirmed in writing the policy of the General Data Protection Regulation (GDPR).

The variables of the study were as follows:

Sociodemographic: age, sex, educational level, employment situation, type of coexistence, place of residence.

Personal survey on coexistence, positive and negative aspects, fears of getting sick or getting infected, and personal reflections on the COVID-19 pandemic.

Psychological and emotional variables related to compassion and self-compassion.

Quality of life related to general health, physical function, physical role, emotional role (anxiety and depression), social function, body pain, vitality, and mental health.

Symptoms of physical fatigue, cognitive and psychosocial fatigue.

### 2.3. Evaluation Instruments

Five scales validated in the Spanish population were used:

1. Quality of Life Scale SF-36 [29]. This is a generic scale that provides a health status profile, and it applies to both patients and the general user population. It is made up of 36 items that analyze the 8 dimensions of health status. Briefly, 1. physical function; 2. physical role; 3. body pain; 4. general health; 5. vitality; 6. social function; 7. emotional role; 8. mental health. The questionnaire allows the calculation of two summary scores, the physical component summary (PCS) and the mental component (MCS). It is useful for evaluating health-related quality of life (HRQoL) in the general population and specific subgroups, comparing the burden of various diseases, detecting health benefits produced by a wide range of treatments, and assessing the individual health status of patients. It has good psychometric properties that have been evaluated in numerous articles and allow the comparison of results; therefore, it is considered one of the instruments with the greatest potential in HRQoL that has been validated in the Spanish population [29].

2. Self-Compassion Scale (SCS) [30]. The SCS questionnaire has 26 items and assesses six factors—the three main factors of compassion and their respective opposite constructs: kindness and self-judgment, common humanity and isolation, and mindfulness and over-identification. This questionnaire offers both a separate score of each component and a total score. It evaluates the extent to which the participants show they are self-compassionate; how they accept that suffering, failure, and defects are inherent to the human condition. Thus, it analyzes the openness towards their suffering, experiencing feelings of kindness towards oneself, with a position free of negative judgments in the face of suffering. The Spanish version [31] of its short form, Self-Compassion Scale-short form (SCS-SF) [32], is reduced to 12 items and was also used in this study. The SCS-SF presents appropriate psychometric properties as Cronbach’s alpha of 0.85, test–retest reliability of 0.89, and high correlations with the long form.

3. Compassion Scale (CS) [33]. The Compassion Scale has 24 self-reported items, and it is based on Neff’s Self Compassion Scale [30] in the assessment of compassion toward oneself (self-compassion) and compassion toward others. The SCS and the CS have the same three main components (kindness, common humanity, and mindfulness), but they differ in their opposites. Thus, in the CS questionnaire, the opposite of kindness is indifference, the common humanity is separation, and mindfulness is the opposite of disengagement. The CS scale was not significantly correlated with Neff’s SCS. The Spanish version of CS [34] has adequate validity and internal consistency (Cronbach’s alpha = 0.89).

4. Modified Fatigue Impact Scale (MFIS) [35,36]. The MFIS is a multidimensional scale, a method widely used to assess fatigue in patients with MS. It is a modified version of the Fatigue Impact Scale (FIS 8) [37]. It has been shown to be an adequate measure of response to change and presents validity for the subjective daily experience of fatigue, originally developed to assess the effects of fatigue on the quality of life of patients with chronic diseases. It has shown reliability and validity in application to different populations of different cultures [38]. It comprises 21 items with high inter-element correlations distributed in 3 subscales: physical, cognitive, and psychosocial. It contains 9 items that measure physical factors, 10 items measuring cognitive factors, and 2 items measuring psychosocial factors. The global scores range from 0 to 84. A cut-off point (38) has been established to define the presence or absence of fatigue. Higher scores indicate a greater impact of fatigue from the disease and on the patient’s quality of life.

### 2.4. Statistics

Statistical analysis was performed with SPSS 25.0 software (IBM Corp., Armonk, NY, USA). First, sample descriptives were made using relative and absolute frequencies, dispersion, and central tendency: mean, median, standard deviation, minimum, maximum, confidence interval (CI), and the number valid of cases. To determine the relationship between categorical variables with two levels and quantitative variables, the Student *t*-test or analysis of variance was used if the quantitative variable assumed normality. The normality of the contrasted variables was evaluated with the Kolmogorov–Smirnoff test. In another case, the non-parametric Mann–Whitney U or Kruskal–Wallis H tests were used. In a second stage, to analyze the correlations between the different quantitative constructs, a Spearman Rho non-parametric correlation matrix was calculated, which included the constructs or psychological variables of self-compassion and the variables related to general health and fatigue. In a third stage, to determine the weight of the constructs of self-compassion and compassion related to physical and emotional health in the measured prognostic variables, a logistic regression analysis was used to control general health and fatigue variables.

## 3. Results

### 3.1. Sociodemographic Descriptives of People with MS and Caregivers

The descriptives of a total sample of the 68 participants (44 people with MS and 24 caregivers) in this study are detailed in Table 1.

Gender and age: Forty-four people with MS voluntarily participated in this study, in a female:male ratio of 70:30 (31 women, 13 men), with a mean age of 49.98 years (95% CI: 47.31–52.65; SD: 8.78, minimum and maximum value of 27 and 70 years, respectively). By age ranges, the highest percentage appeared in the age group between 46 and 55 years. Twenty-four caregivers voluntarily participated, in a female:male ratio of 67:33 (16 women, 8 men), with a mean age of 45.90 years (95% CI: 39.53–52.30, SD: 14.76, minimum and maximum value of 23 and 67 years, respectively). By age ranges, the highest percentage also appeared in the age group between 46 and 55 years. The distribution by age bands of the samples of MS patients and MS caregivers is shown in Table 1.

Marital status: Almost 80% of the MS patients were married or with a partner. The rest of the sample was distributed among singles (13.6%), separated and/or divorced (6.8%). These last two categories accounted for approximately 20% of the sample. Of the MS caregivers, 70.8% were married or with a partner, and the rest were single (29.1%).

Living arrangements: Regarding residence, 98% of the MS patients and MS caregivers resided in Teruel and the province and 2% in Zaragoza. About half of the MS patients (43.2%) lived in their own home with a partner and/or children, followed by those who lived with a partner without children (34.1%), while 13.6% lived in the home of relatives, and 9.1% lived alone in their own home. Practically half of the MS caregivers (45.8%) lived in their own home with their partner, and 33.3% lived with their partner and children; 8.2% lived at the home of relatives or neighbors.

Education: Regarding the academic level of MS patients, 50% had higher university studies, almost 30% studied until secondary school, 18.2% had completed primary studies, and only 2.3% had done no study. Almost 80% of MS caregivers had attended secondary school and/or university, and 20.8% had only primary education.

Employment situation: Retirees accounted for 29.5% of the MS patients. The next largest group was people with permanent disabilities (27.3%), and 22.7% were working; 11.4% were self-employed and/or entrepreneurs, 4.5% were housewives or dedicated to caring for the family. Just 4.6% were unemployed with temporary leave. The employment situation of MS caregivers was as follows: 45.8% were working, 16.6% were students, and 12.5% had sick leave and retired, respectively.

### 3.2. COVID-19, Confinement and Fears

**Figure 1:**
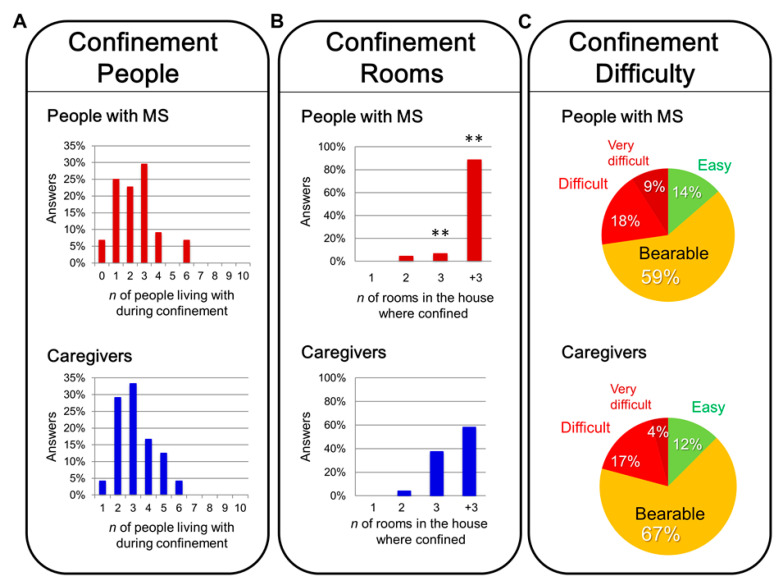
Confinement–people, rooms, and difficulty. Sample distribution of the people with MS and caregivers answering to the number of people living with them during the confinement (**A**), the number of rooms of the house where they were confined (**B**), and the level of difficulty they had handling the period of confinement (**C**). Statistics: Fisher’s test, ** *p* < 0.01 People with MS vs. caregivers.

**Figure 2:**
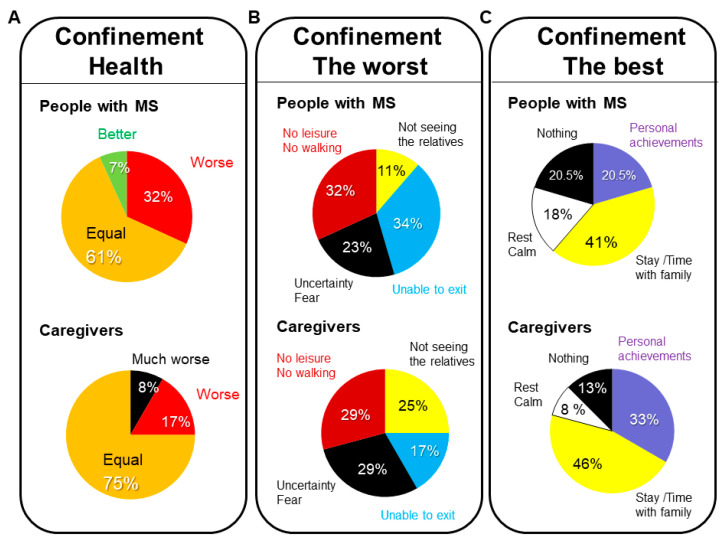
Confinement–health, the worst, and the best. Sample distribution of the people with MS and caregivers answering to their health status as compared to before the confinement: (**A**) the worst of the confinement (**B**), and the best of the confinement (**C**).

**Figure 3:**
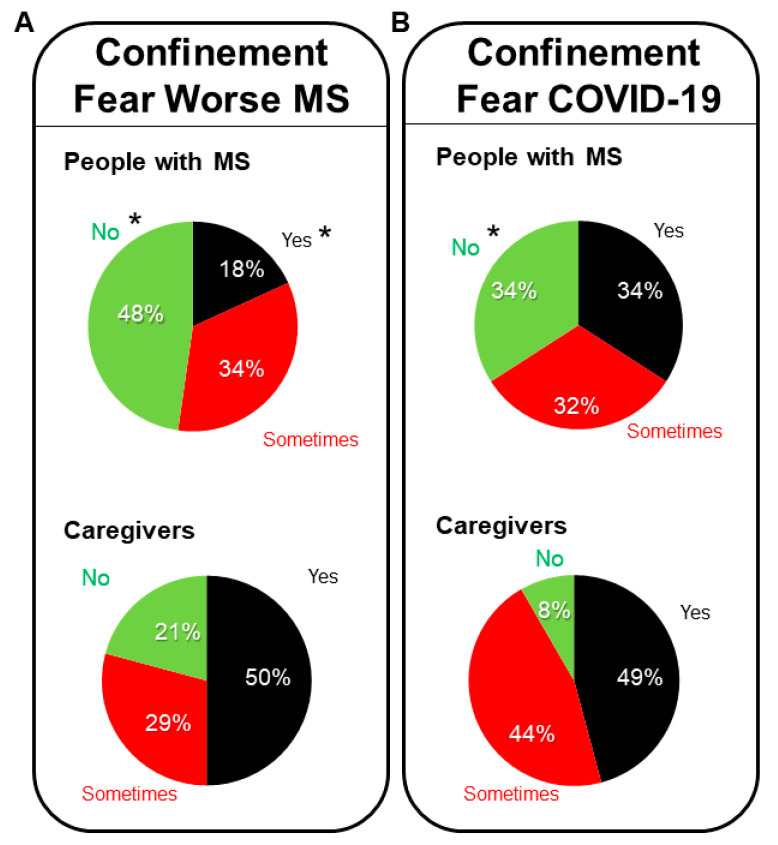
Confinement–fears of worsening of MS and COVID-19 related fears. Sample distribution of the people with MS and caregivers answering on their fears of MS being worsened during the confinement (**A**), and fears about getting infected and ill with COVID-19 (**B**). Statistics: Fisher’s test or Chi-square, * *p* < 0.05 people with MS vs. caregivers.

Figure 1, Figure 2 and Figure 3 illustrate the features of the confinement for the sample population. During their confinement, MS patients were living in a home (Figure 1A) with a 2 to 4 person structure (22% with another person, 20% with 2 people, 29.55% with 3 people). In the case of caregivers, the structure was of 3–4 people at home (29.17% with 2, 33.33% with 3). Most answers about the number of rooms during confinement (Figure 1B) were “more than 3 rooms” in both groups, but the frequency was higher in MS patients (88.64% vs. 58.33%, Fisher’s test, *** *p* = 0.0062) since few of their homes had only 3 as compared to caregivers (7% vs. 38%, Fisher’s test, *p* = 0.0026). Referring to how both groups have coped with confinement (Figure 1C), 59.09% of MS patients and 66.67% of caregivers rated it as “bearable.” There were similar scores for “relatively easy” (MS, 64% vs. caregivers, 12.50%) and “difficult” (MS, 18.18% vs. caregivers, 6.67%). A total of 9.09% of the MS patients considered confinement “very difficult” to handle, while only one caregiver (4.17%) referred to it so.

When asked about their current health (Figure 2A) as compared to that they had before confinement, the highest scores were obtained for “equal” in both groups (MS, 61.36%; caregivers 75.0%). However, the answer “worse” or “much worse” was given by more MS patients than caregivers (MS, 31.82% and 2.3%; caregivers, 17.4% and 8.7%, respectively). Three patients considered their health was better or much better than before, while none of the caregivers chose these positive options.

In the open questions on the worst of confinement (Figure 2B) “unable to exit” was the worst for 34.09% of the MS patients, while “not having leisure–walking” was referred to in 31.82% of comments. For caregivers, “not having leisure–walking” and “uncertainty–fear” were the most expressed concepts (29.17% each).

On the positive side, the best of confinement (Figure 2C) for the MS patients was “being with the family or having more time with them” for 40.91%, and it was also the most common benefit referred to by caregivers (45.83%). On the other hand, one (20.45%) patient and one (12.50%) caregiver answered with “nothing”.

Concerning fear that MS will worsen during the pandemic (Figure 3A), 47.73% of the people with MS answered with a “no” as compared to 52.27% who affirmed that “yes” they were afraid (18.18%) or “sometimes” (34.09%). For their counterparts, the caregivers, half of them answered “yes” (50%) and 29.17% “sometimes”, as compared to 21.1% who said “no”. Thus, statistically significant differences were found with regards to this fear, with fear of worsening of MS being higher among the patients than in the caregivers’ group (Fisher’s test, * *p* = 0.0379), and a significantly lower number of people with MS affirming to be worried about it than their caregivers (Chi-square, 6.118, 1df, * *p* = 0.0134).

When individuals were asked if they were afraid of getting infected and sick with COVID-19, 34.09% MS patients answered both “yes” and 34.09% “no”. In contrast, caregivers were more afraid, as only two of them (8.33%) answered negatively, and the other 22 (91.7%) were afraid, with 48.83% affirmative “yes” or 43.5% “sometimes”.

### 3.3. Fatigue, Self-Compassion, and Compassion in People with MS and Caregivers

Table 2 and Table 3 depict the analysis of the dimensions of the perception of quality of life related to the physical and emotional health of MS patients in the last four weeks of June and early July 2020 post-confinement. On average, values of moderate health were found in the state of general health, physical function and role, body pain, vitality, social function, emotional role, and perceived mental health. The data also indicated that the physical health of these patients had not interfered with their daily activities and their perception of health. This also included their prospects and resistance to getting ill in these times of the COVID-19 pandemic that presented moderate to average levels.

The results obtained on the assessment of mental health related to anxiety, depression, and self-control remained within normal values of mental well-being. However, the self-compassion scores of MS patients presented a medium–high level. Based on the cut-off points, it was found (Table 2) that there were high fatigue levels, mainly physical and cognitive fatigue. However, psychosocial fatigue remained at a medium level. The great dispersion obtained in the fatigue results (DT 8.99 and DT 9.6, respectively, in the group of people with MS) should be noted.

The caregivers of people with MS obtained high scores in compassion, as well as in their health levels and quality of life related to a good perception of health and to their physical function, vitality, mental, and emotional health. On the other hand, the scores related to fatigue were lower than the cut-off point, which indicated the absence of physical, cognitive, or psychosocial fatigue. However, a great dispersion in the results should also be noted (DT 8.6; DT 8.7) in this group.

### 3.4. Correlations between Self-Compassion in People with MS and Compassion in Caregivers with Physical and Mental Health, including Fatigue

The analysis of the psychological variables of self-compassion and compassion and the different physical and mental health variables, including fatigue, and a bilateral correlation with Spearman’s coefficient is depicted in Table 4.

The correlations between self-compassion in the people with MS and the scales of physical role, social function, vitality, and global health were positive and statistically significant (** *p* < 0.01, * *p* < 0.05). A negative correlation was found between global fatigue and cognitive fatigue. The impact of fatigue (cognitive and global) in people with MS showed significant correlations with the psychological variables of self-compassion.

The compassion scale completed by caregivers did not show a significant correlation or association with the variables related to mental, physical, or global health, nor fatigue.

### 3.5. Regression Analysis of Self-Compassion in People with MS, and Compassion in Caregivers

Two regression analyses were carried out, considering the criteria variables, the global fatigue and the perception of global health, and as explanatory variables, the psychological variables of self-compassion mentioned (Table 5). In the final model, self-compassion was maintained as an explanatory factor in 19% of the global health variance in people with MS (Table 5). The regression analysis was also performed on the sample of caregivers, considering global health and fatigue as the dependent variables and compassion as the independent and explanatory variable. However, the study results did not reveal that compassion was a predictive/explanatory factor for overall health and fatigue in the sample of caregivers.

## 4. Discussion and Implications

### 4.1. Sociodemography, COVID-19 Confinement and Fears

The COVID-19 pandemic lockdown, with the paralysis of economic activity, the closure of educational centers, and the confinement of the entire population for weeks were extraordinary situations and multiple stress-generating stimuli at the societal and individual level that persist today. Home confinement is an unprecedented situation that has implications for people’s physical and psychological well-being [39]. The sociodemographic status and cultural background can make a difference in the impact of these stressors associated with the COVID-19 pandemic, mainly in those who already have a disease condition, their caregivers, and their families. In the present work, the status of the people with MS was good enough to provide a ‘sociodemographic’ resistance to the individuals, helping to mitigate the effects of lockdown. Family structure at home was 2 or 3 people, in houses with good living space (more than three rooms). From a gender perspective, ratios in the sample of participants with MS showed an overrepresentation of females, in agreement with the general prevalence of the disease with a 65:35 female: male ratio, which is on the increase [18,40,41]. In a recent online survey by Zhang et al. [42] of people with MS from Sevilla, a southern Spanish area, and several provinces of China, including a high proportion of young patients, no differences in self-reports on social and work effects of confinement were identified regarding sex. In their work, despite Spanish people with MS having greater economic stability and social support, similar perceptions of the pandemic’s social and work consequences were reported, with the use of social networks and family support being also similar in both groups.

To our knowledge, the present work is the first to analyze the effect of confinement on both people with MS and their caregivers, providing a bio-psycho-social approach. In agreement with the traditional gender of those dedicated to caring for older people or the sick in familialist countries like Spain and Italy, caregivers were also predominantly female [43,44].

According to the mean age of the sample and their marital status, and considering that MS mainly affects young subjects, we can infer that MS was experienced in its chronic nature in most participants. This is also important to consider regarding the open questions about the best/worst of the confinement since the patients’ physical limitations and fatigue impede their quality of life.

Despite more patients considering confinement more difficult than caregivers, they were less afraid of worsening MS or afraid of getting COVID-19 but recognized the worsening of their heath during the strict lockdown. The reference to the inability to exit and not do their regular walks and rehabilitation programs as the worst of the confinement warns about the impact that the restrictive conditions had in their lives. Therefore, efforts to implement home-based interventions are strongly needed. For instance, a recent systematic review shows the positive effects of home-based active video game interventions on gait and balance in persons with multiple sclerosis [45]. Despite this being easily predicted to affect physical health, it is noteworthy that lack or substantial reduction of physical activity and contact with the natural environment may have prevented MS patients and caregivers from receiving its beneficial effects on mood. The impact of the constraints on the mental health of patients and caregivers should not be underestimated. Conversely, a recent report showed that moderate-intensity physical activity was an effective strategy to modulate emotional distress during the COVID-19 pandemic in a population of working mothers experiencing heightened levels of parenting stress [46].

As resistance/resilient aspects, both patients and caregivers agreed that “staying with or time with the family” was among the best effects of the confinement. Although these spontaneous answers were also expected, these statements are supported by cultural and traditional roots since long-term care models in Spain and Italy are usually labeled familialist or family-based [43,44]. Although the worst of lockdown was reclusion and lack of walks, the caregivers also referred to lack of leisure and uncertainty–fear, which would fit with caregivers’ compassionate role. This also fits with their fears about the worsening of the MS, or themselves getting infected and ill from COVID-19 and the associated risk of death. Pessimistic answers in some patients and caregivers were also noted when they answered with a simple ‘nothing’ to what was ‘the best’. Depressive symptoms, loneliness, insomnia, and daily life fatigue have been reported as common disruptions of mental well-being during COVID-19 confinement [7,8,9,10,11,12]. A prospective cohort study by Andreu-Caravaca et al. [47] conducted in Murcia, a middle-eastern Spanish area, investigated the effects of home confinement on low sleep quality, cardiac autonomic dysfunction, and poor quality of life. These are some of the most prevalent symptoms in people with MS and are known to worsen with the progress of MS and, most importantly in the current context, with physical inactivity. Their results showed that the strict confinement worsened sleep quality, but not their cardiac autonomic control as measured by heart rate variability, nor their health-related quality of life. The worsening of these three variables is among the major-stress related symptoms reported during lockdown with sleep disorders (so-called coronasomnia) as one of the most prevalent in this COVID-19 scenario [7,8,9,10]. Considering these and other aspects during the period of confinement, Reguera-García et al. [48] conducted a study on 84 people with MS belonging to MS associations in Castilla and Leon, a northwest area of Spain. Their work reports average levels of a ‘sense of coherence’ (SOC-13), high levels of ‘resilience ‘(ER-14), and also of ‘coping’ (COPE-28), primarily through active confrontation and religion. These psychological variables were correlated among them but were not related to the moderate to high score levels of physical activity (IPAQ-SF) recorded in this sample population.

### 4.2. Fatigue, Self-Compassion, and Compassion in People with MS and Caregivers

In this study, the impact of fatigue on the physical, cognitive, emotional, and psychosocial dimensions was analyzed. The fatigue levels evaluated in people with MS and caregivers showed similar results to those reported in a cohort of 227 patients with MS where fatigue was a persistent symptom during the 18-month duration of the study in 86.8% of participants [49]. In another longitudinal study on a population of 267 patients with MS, where fatigue severity was assessed three times a year, 38% had persistent fatigue (FSS > 5 on all 3 occasions), 37% had sporadic fatigue (FSS > 5 on 1 or 2 occasions), and 25% did not have fatigue [50].

Self-compassion was evaluated in people with MS in times of the COVID-19 pandemic in Teruel during the months following the confinement in June–July 2020, and the results showed a high score for self-compassion. In the Buddhist philosophy, self-compassion postulates that suffering is a natural and inevitable condition in human beings. Therefore, it is necessary to tune in to it and have the predisposition to alleviate it [51]. Furthermore, scientific evidence about the psychological aspects, both functional and dysfunctional, is associated with the construct of self-compassion [52]. In the healthcare setting, compassion allows emotional commitment to patients and their needs [53]. Some studies show that the experience of “self-compassion” protects against stress and anxiety, eliminating thoughts that decrease self-confidence [54]. Self-compassion also helps to connect with negative emotions and develop strategies to address difficulties, take responsibility for negative events, and foster an internal dialogue with kindness and understanding of the personal deficiencies that cause suffering [55].

The present results in people with MS highlight the relevance of self-compassion in their lives in relation to social variables and quality of life variables related to their health in terms of fatigue, physical role, energy, or vitality. This ability to be self-compassionate can be an important source of resistance and resilience since it implies accepting that suffering, failure, and one’s own defects are part of the human condition, evaluating openness towards one’s own suffering, and experiencing feelings of kindness towards oneself, with a posture free of negative judgments in the face of suffering, as described by Neff [30]. The present results showed that a higher score in self-compassion correlated positively with the variables of social function, vitality, and physical role, and negatively with cognitive fatigue. In contrast, compassion did not. Most importantly, the self-compassion construct was found to have explanatory and predictive validity concerning the HRQoL (health-related quality of life) in the sample of people with MS in the Teruel Study. That is, global health involved the constructs of physical function, physical role, body pain, and vitality for general health, but also the emotional role and social function for mental health. Therefore, according to Yarnell et al. [56], if self-compassion is an ability to open up to the experience of pain without reacting to it, we will have to delve into the study of this construct and see the influence and impact of the social environment to which the individual belongs.

Consistent with the results of our study, there is scientific evidence on the benefits of self-compassion. Self-compassion has been associated with physical and emotional well-being, emotional intelligence, social function, satisfaction with life, feelings of competence, happiness, optimism, and knowledge. Self-compassionate people adhere better to diet treatments, exercise, and anti-tobacco addiction programs, and reduce procrastination. Self-compassion has also been associated with a decrease in anxiety and depression. It should be noted that self-compassionate people improve their self-esteem, but it does not depend on comparing themselves with others. People who score high on self-compassion find a way to comfort themselves when they fail by learning and growing from their mistakes in a resilient way. They are able to motivate themselves instead of criticizing themselves [15].

According to Gilbert et al. [57], with reference to self-compassion, he discovered that different subsystems of the nervous system are activated when we are compassionate and when we feel threatened. When the threat system is activated in a particular way by challenging situations, where sadness, anger, or fear appear, we likely attack ourselves, abandon ourselves, or block ourselves in anxious worry. However, when the compassion system is activated, the body interprets it as a security signal; we console ourselves, encourage ourselves, and relate kindly to ourselves. This research showed that different brain areas are activated when being self-critical and self-compassionate [58]. Therefore, the interpretive process, partly at the unconscious level of the patient with MS, could explain the ability to protect and give security to their immune system already damaged by MS and not harm it to a greater extent due to their negative emotions.

Rogers [59] also referred to the relevance of the paradox of life: when a person accepts him/herself as he/she is, he/she can change. Research has shown that the more we push ourselves, or beat ourselves up trying to improve, the harder it is to make a change. Self-compassion is one of the foundations of change. In the present work, the sample of people with MS had moderate scores on self-compassion and were more likely to learn from their mistakes and recommit to their goals. Gilbert et al. [60] also showed that people who suffer from depression and anxiety realize that their thoughts are irrational, but they cannot stop thinking about them. MS patients did not score high levels in distress, anxiety, and depression; therefore, it is expected that they have changed the tone of their conversations when talking about their disease and established cordial relationships. They have their emotional pain but share it with others from a more caring and healing, as well as comforting position, for the collective of the association to which ATUEM belongs. The critical role MS networks had during the strict confinement was also referred to in the Castilla-León study [47], and it is always implicit in the existence of these associations where social understanding and support are so needed.

Compassion has been approached from different areas such as philosophy, positive psychology, social, neuropsychology, pedagogy, and spirituality, both in the East and in the West [61]. Lazarus and Lazarus [62] stated that compassion involved understanding the emotional state of others and that it was associated with a desire to alleviate or reduce the suffering of another person [63]. Compassion is considered an affective experience related to emotions, moral values, judgments about oneself and towards others, and is also associated with personal and social well-being and depends on the cultural and social context evaluated [64,65]. However, in the present study, the results of the caregivers of people with MS did not reveal information and did not show an association of the compassion construct with their quality of life related to their physical and emotional health. Our study hypothesized an association between the compassion and self-compassion constructs and the predictive and explanatory capacity of health outcomes, but the results address different outcomes from those contemplated by the current literature.

Within the adversities, unfavorable conditions, and multiple mourning processes with sudden and massive loss of human lives in the current COVID-19 pandemic, psychotherapeutic interventions based on self-compassion and compassion would be indicated in these particular psychological and medical conditions of people with neurodegenerative diseases, including patients with MS. In addition, these interventions can be developed, operationalized, and improved through practice. According to Kirby [66], there are programs based on compassion with empirical evidence, such as compassion-focused therapy [67], and mindful self-compassion training [68]. Following the proposal of Strauss et al. [69] and after the results of the present study, one of the lines of psychological approach would be to conceptualize compassion as a cognitive, affective, and behavioral process that would enhance five elements referred to both as self-compassion and compassion for others: (a) being aware of suffering, (b) assuming it as a universal phenomenon in the human condition, (c) showing empathy for those who suffer and connecting with their emotions, (d) being tolerant of the annoying feelings that are aroused by the response (anguish, anger, fear) by remaining open and accepting the sufferer and (e) being motivated to act and alleviate suffering.

Developing compassion and self-compassion entails enhancing other psychological variables and favors interpersonal relationships and their social function, such as empathy, sympathy, love, altruism, prosocial behaviors, and feelings of pity [59]. Sensitivity also appears as the ability to respond to the emotions of others and to be able to perceive the need for care from others, the motivation to act or respond to the suffering of others, tolerance to anxiety, and the ability not to judge, since they are relevant, as the authors emphasized that compassion consists of helping others without overidentifying with their suffering and without judging them. In this way, we can feel compassion for someone we do not like or have no affinity with [47].

## 5. Limitations

The study’s limitations include the inherent cultural and social factors and the size of the sample population. Due to the difficulties of recruiting people with MS but primarily caregivers in the chronological scenario, the study does not allow us to generalize the results to the general population with MS. Since the impact of confinement would strongly depend on the living conditions and the demographic structure of the cities, we decided to choose the people with MS of ATUEM, which gathers together patients from a high-altitude and small 14,809 km^2^ area in a mountainous region of eastern Spain, with a humid subtropical, bordering on a cold semi-arid, climate. Of its 134,572 (2018) population, about a quarter live in Teruel, the capital, with a low population density, making it the least populated provincial capital in the country and providing a more homogeneous sample at the demographic, cultural, and environmental level. However, as discussed, similarities have been found in the works carried out by MS associations. Through the different approaches and topics addressed together, a complete picture of the effects of strict confinement in people with MS can be offered to gain knowledge in this respect that will be helpful for better preventive/therapeutic interventions and decision-making in public health. Most importantly, follow-up studies will be necessary for disease monitoring, including both bio-medical and medical psychology models, and to provide better support to caregivers.

## 6. Conclusions

The results of a survey of MS patients and caregivers concerning COVID-19 confinement and their fears indicated that despite living in better housing conditions, more patients considered confinement more difficult than caregivers. Surprisingly, they were less afraid of worsening MS during the strict confinement, probably due to acceptance and self-compassion. Still, the patients recognized worsening of their health status as compared to before the pandemic. The worst of lockdown was reclusion and lack of walks. For the first time, in this scenario, caregivers’ reports were recorded parallel to the people they cared for. They also referred to lack of leisure and uncertainty–fear. Both groups agreed that the best was ‘staying with the family or having more time with it,’ but some complained, reporting a simple ‘nothing’.

In the second part of the study, the answers of patients and caregivers to the psychological tests indicated that:

(1) Self-compassion evaluated after the post-confinement in the months of June–July 2020 of the MS patients remained at a moderate–high level.
(2) MS patients perceived their physical and emotional health during June–July 2020 as at medium and moderate levels.
(3) The fatigue of MS patients during June–July 2020 presented high scores, mainly in physical and cognitive fatigue.
(4) The self-compassion of the group of MS patients significantly correlated with fatigue and global health (physical and emotional) and presented explanatory validity with a 19% variance of global health.
(5) The high compassion of the caregivers of MS patients did not show relationships with any physical or emotional health variable, nor with fatigue scales.

Overall, this study reveals the importance of the self-perception of these patients towards their neurodegenerative disease that inherently implies severe physical, psychological, and social stress. It also analyses the explanatory capacity of the factors that promote self-compassion, the patient’s own commitment response to alleviate suffering. It has also revealed psychological variables and coping styles that can limit or hinder the expression of the human capacity in dealing with the disease in all its physical and psychological aspects.

The answers to the above questions, depending on the respondents’ personal, family, social and professional situations in the middle of the COVID-19 pandemic and asked after the most extraordinary period of strict confinement that they had ever experienced, allow this study to be useful and clinically relevant to envision the impact of the pandemic and the design of the post-COVID era for these patients and their caregivers.

## Figures and Tables

**Table 1:** Distribution of the samples of people with MS and their caregivers according to sociodemographic variables.

		People with MS		Caregivers	
		*N* = 44		*N* = 24	
		Frequencies	%	Frequencies	%
Gender	Female	31	70.45	16	66.67
	Male	13	29.55	8	33.34
Age	<36 years	2	4.55	7	29.16
	de 36 a 45	10	22.73	2	8.34
	de 46 a 55	21	47.73	9	37.5
	>55 years	11	25.00	6	25
Marital status	Married/living with partner	35	79.55	17	70.83
	Single	6	13.64	7	29.17
	Separated/divorced	3	6.82	-	-
Living arrangements	Lives alone	4	9.09	3	12.5
	Lives with partner/spouse	15	34.09	11	45.84
	Lives with partner/spouse and children	19	43.18	8	33.34
	Lives with other family	6	13.64	1	4.16
	Other	-	-	1	4.16
Education	No qualifications	1	2.27		
	Primary school	8	18.18	5	20.83
	Secundary school	13	29.55	9	37.5
	University	22	50.00	10	41.67
Employment situation	Student			4	16.67
	Homemaker	2	4.55	1	4.17
	Unemployed	1	2.27	-	-
	Employed	10	22.73	11	45.83
	Temporary unemployed	1	2.27	3	12.5
	Retired	13	29.55	3	12.5
	Permanent disability	12	27.27	1	4.17
	Other	5	11.36	1	4.17

**Table 2:** Descriptive statistics of self-compassion, health, and fatigue variables in people with MS.

People with MS (*N* = 44)	Mean	Median	SD	Range	Min	Max	95% (IC)
Self-Compassion	3.22	3.16	0.565	2.08	2.25	4.33	3.05–3.39
Physical function	20.9	22	5.838	20	10	30	19.13–22.68
Physical role	5.79	5.5	1.636	4	4	8	5.29–6.29
Emotional role	5.36	6	1.122	3	3	6	5.02–5.70
Social function	7.97	8	2.085	8	2	10	7.34–8.61
Body pain	7.72	8	2.433	8	3	11	6.98–8.46
Vitality	13.9	15	4.917	17	5	22	12.41–15.40
General health (PCS)	17.18	17	3.642	14	10	24	16.07–18.28
Mental health (MCS)	23.2	23	3.825	15	15	30	22.04–24.36
Global health (HRQoL)	102.1	103	17.775	63	68	131	96.66–107.47
Physical fatigue	20.18	23	8.994	34	0	34	17.44–22.91
Cognitive fatigue	15.06	18.5	9.604	33	0	33	12.14–17.98
Psychosocial fatigue	3.56	4	1.921	8	0	8	2.98–4.15
Global fatigue	38.81	43.5	17.838	61	0	61	33.39–44.24

Confidence interval = 95% (IC); Min = minimum; Max = maximum; SD = Standard deviation.

**Table 3:** Descriptive statistics of compassion, health, and fatigue variables in MS caregivers.

MS Caregivers (*N* = 24)	Mean	Median	SD	Range	Min	Max	95% (IC)
Compassion	3.84	3.9	0.442	1.5	2.96	4.54	3.65–4.03
Physical function	26.13	28	4.902	16	14	30	24.01–28.25
Physical role	6.78	8	1.565	4	4	8	6.10–7.45
Emotional role	4.83	5	1.267	3	3	6	4.27–5.37
Social function	7.13	7	2.262	8	2	10	6.15–8.10
Body pain	8.48	9	2.644	9	2	11	7.33–9.62
Vitality	14.65	15	3.961	14	7	21	12.93–16.36
General health (PCS)	20.35	21	4.323	17	10	27	18.47–22.21
Mental health (MCS)	20.52	22	5.265	22	7	29	18.24–22.79
Global health (HRQoL)	108.87	117	21.808	86	52	138	99.43–118.29
Physical fatigue	11.82	9	8.680	32	1	33	8.06–15.58
Cognitive fatigue	11.91	10	8.789	30	0	30	8.11–15.71
Psychosocial fatigue	2.86	2	2.399	7	0	7	1.83–3.90
Global fatigue	26.60	23	18.376	64	5	69	18.66–34.55

Confidence interval = 95% (IC); Min = minimum; Max = maximun; SD = Standard deviation.

**Table 4:** Correlations between psychological variables of self-compassion and compassion and variables of health and fatigue in a sample of people with MS and their caregivers (*rho* Spearman method).

	*rho* Self-Compassion	*rho* Compassion
Physical function	0.155	0.247
Physical role	0.330 *	−0.011
Emotional role	0.191	−0.054
Social function	0.387 **	−0.052
Body pain	0.269	−0.089
Vitality	0.456 **	0.084
General health (PCS)	0.296	0.328
Mental health (MCS)	0.278	0.134
Global health (HRQoL)	0.436 **	0.170
Physical fatigue	−0.285	0.023
Cognitive fatigue	−0.380 *	0.132
Psychosocial fatigue	−0.262	−0.036
Global fatigue	−0.455 **	0.040

PCS, physical component summary; MCS, mental component summary; HRQoL, health-related quality of life; *rho* = *rho* Spearman; * *p* < 0.05; ** *p* < 0.01.

**Table 5:** Regression analysis of self-compassion on the global health assessed as HRQoL (health-related quality of life) in people with MS. Stepwise method.

Model	R	R^2^	R^2^ Adjusted	ESE	Chance R^2^	F-Chance	gl1	gl2	*p*-Value
1	0.441	0.194	0.175	16.144	0.194	10.129	1	42	0.003 **

Dependent variable: global health (HRQoL). Predictors in the model: constant and self-compassion ESE: estimated standard error, ** *p* < 0.01.

## Data Availability

Not applicable.
